# Common Nasal Conditions as the Cause of Neuralgias of the Terminal Branches of the Fifth Nerve*Read at Bryan, Texas, November 12, before the Brazos Valley Medical Association.

**Published:** 1902-03

**Authors:** Joseph Mullen

**Affiliations:** Houston, Texas


### TEXAS MEDICAL JOURNAL.

ESTABLISHED JULY, 1885.

PUBLISHED MONTHLY.—SUBSCRIPTION $1.00 A YEAR.

VOL. XVII. AUSTIN, MARCH, 1902. No. 9.

### Original Contributions.

For Texas Medical Journal.

### Common Nasal Conditions as the Cause of Neuralgias of the Terminal Branches of the Fifth Nerve.*

BY DR. JOSEPH MULLEN, HOUSTON, TEXAS.

The extensive prevalence of neuralgias of the terminal branches of the fifth nerve, and their seeming intractability to general treatment, afford a sufficient excuse for presenting this interesting and important every day subject. Neuralgias of these branches are the commonest of all conditions that we, as physicians, are called to treat. It is a complaint of all ages, sexes and conditions, and the one which is treated the most generally by the laity without regard to cause, duration or results. There is hardly a gentleman present but who can recall cases of chronic headaches, that have begun treatment by taking some proprietary preparation and finally ended by becoming a morphinomaniac,—hence this attempt to simplify and differentiate the various common nasal causes of neuralgias of the fifth nerve from those due to the eyes, stomach, etc.

The location of the neuralgia may be in the eyeball, passing from the nose through the ophthalmic ganglion; in the supra- or infra-orbital ridge coming as a direct nerve reflex from the nasal

*Read at Bryan, Texas, November 12, before the Brazos Valley Medical Association.

branch of the ophthalmic division of the fifth, or it may be felt in the teeth of the upper jaw, through the superior maxillary division of the nerve. Again the seat of pain may be in the ear or temple, reflexly transmitted through the auricular and temporal branches. The seat of pain may also be found very severe in character over the orbital plate of the ethmoidal bone, or directly over the nasal bones. The frontal region may also be referred to as painful. This pain is probably dependent upon vernous stasis or engorgement of the nasal plexus of veins.

The pain may vary from a feeling of uneasiness to that of the most excrutiating, producing temporary loss of consciousness. It may be temporary in duration, remaining only a short time, clearing up with physical exercise; or it may remain for days without changing much in character or intensity, responding to no general treatment or medication, except morphine. Then again the patient may be awakened early in the morning, between three and six o'clock, with a headache lasting until the sun comes up strong, and exercise is indulged in, or a hot stimulating drink has been taken. The pain comes on again when the sun goes down, the air becoming damp, to disappear just before going to bed. In the morning the daily cycle begins anew. The pains, however, do not necessarily return every morning, but as a rule they do come as an early morning headache. An opposite recurrence of headache prevails in atropic rhinitis; the dampness of the atmosphere relieves the pain of this disease, felt on the bridge of the nose and in the eye, while the heated portion of the day intensifies or makes it worse. These pains also frequently follow in the wake of an acute rhinitis, at the stage when all the acute symptoms have passed away, and the thick catarrhal secretions have ceased; in other words, the terbinal membrane fails to return to the normal, when the swelling and engorgement increases, producing nasal pressure. I have known of many cases of pregnancy in which nasal reflex pains were present; where the pains began about the second month of pregnancy, and lasted until the sixth month, and then cleared up. All the cases had a previously existing hypertrophic rhinitis, and hence were very susceptible to acute cold in the head. The recurrence and production of reflex nasal headaches are not brought about by the use of the eyes, and rarely come on after 10 a. m. and before 5 p. m., unless the patient has an acute coryza, or the day is a cold or rainy one. While headaches from eye strain follow prolonged use of the eyes, and are relieved by sleep and a good night's rest, on the other hand nasal headaches come on early in the morning after a night's rest, and disappear as the day becomes warmer, or it is frequently relieved by physical exercise, more so than eye headaches. Nasal headaches are also made better by eating a hearty meal or drinking alcoholic stimulants. The commonest of all nasal causes of neuralgias of the terminal filaments of the fifth nerve, is nasal pressure induced by the commonest of all nasal conditions, hypertrophic rhinitis. This condition is prevalent everywhere and at all seasons of the year, and is the direct outcome of frequent and untreated attacks of coryza. It does not follow, however, that all cases of hypertrophic rhinitis have reflex neuralgias of the fifth nerve. A large proportion of patients show a transient attack of mild pain after an acute coryza has just cleared up. In other cases the pain continues increasing in severity and duration, the membrane seeming not to have the recuperative powers to return to its previous condition. The most common place in the nasal cavities for pressure of this character is first on the middle turbinals, and secondly in the anterior horn of the superior turbinal bone. Nasal spurs are also a frequent cause of nasal pressure, usually involving the nasal process of the vomer. As in the case of hypertrophic rhinitis, there are many cases of septic spurs, which do not produce nasal pressure. I recall a trained nurse whose left eye had been pronounced glancomatous; the pain in the eye had become so intense that she was unconscious, intra-ocular pressure was plus 2; the pupil, however, was markedly contracted, the peri-corneal injection was quite apparent. On examination of the left nasal cavity, it showed pressure of the hypertrophic membrane upon a small nasal spur—this was retracted with cocaine solution and small pledget of cotton interposed with iodine, and in a few moments she was conscious. Nasal pressure does produce conditions other than local pains along the course of the fifth nerve. Epilepsy, chorea, and a number of other diseases may be dependent upon it.

Nasal polypi also frequently produce these headaches; when they do, however, dampness and rain always increase the pains. They are more or less constantly present, but improve somewhat during dry weather.

Diseases of the accessory nasal chambers, such as the frontal, ethmoidal, maxillary and sphenoidal sinuses, are found to frequently cause these headaches. The presence of sinus disease is more easily detected now than formerly, especially since Hajek, of Vienna, has investigated the subject so fully. At times is is quite difficult to detect these conditions, and then only after repeated and careful examination of the most exhaustive character. It is needless to say in the treatment of all the causes of neuralgias of the fifth nerve enumerated that the important consideration is to make a correct and accurate diagnosis, and finally to remove the cause, be it nasal pressure induced by hypertrophic rhinitis, simple nasal engorgement, nasal spurs, or sinus disease.

